# Editorial: DNA Methylation in Plants Associated With Abiotic Stress

**DOI:** 10.3389/fpls.2021.778004

**Published:** 2021-10-18

**Authors:** Markus Kuhlmann, Hua Jiang, Marco Catoni, Frank Johannes

**Affiliations:** ^1^Department Molecular Genetics, RG Heterosis Leibniz Institute of Plant Genetics and Crop Plant Research (IPK), Gatersleben, Germany; ^2^Independent Research Group Applied Chromosome Biology, Leibniz Institute of Plant Genetics and Crop Plant Research (IPK), Gatersleben, Germany; ^3^Research Group Plant Epigenetics and Genome Plasticity, University of Birmingham, Birmingham, United Kingdom; ^4^Department Molecular Life Sciences, Research Group Population Epigenetics & Epigenomics Technical University of Munich, Munich, Germany

**Keywords:** DNA methylation, epigenetics, plants, abiotic stress, RdDM RNA-directed DNA methylation

Methylation of DNA is an evolutionarily conserved modification. It is associated with heterochromatic structures. Together with histone modifications, DNA methylation generates unique patterns that support gene regulation, chromatin structuring, and repression of repetitive elements (Bhadouriya et al.). This modification provides a heritable mark that can be propagated through mitosis and meiosis. The methylated region of DNA is recognized and interpreted an epigenetic toolkit involving readers, writers and erasers. In most higher organisms, DNA methylation is restricted to symmetric cytosines. Due to the symmetry, the pattern can easily be propagated from one cell generation to the next after replication. Plants are the only organisms that display significant methylation of asymmetric cytosines, which represents a unique feature of regulation. This mechanism, defined as RNA-directed DNA methylation (RdDM), involves the presence of small regulatory RNAs as triggering molecules and was reviewed here by Liu and He and Kumar and Mohapatra.

As several of the identified regulatory components of DNA methylation respond to the environmental and developmental conditions (Kumar and Mohapatra), the pattern of methylation in the genome can also change. Some of the environmental changes can occur from minutes to hours, others can affect longer periods like days, weeks, or even years for perennial plants. These changes can result in differential methylated regions in the genome (DMRs). If a DMR is located in the regulatory region of a gene, it might influence transcriptional activity. In several cases, methylation of a promoter element leads to suppress the expression of the associated gene, a phenomenon known as transcriptional gene silencing. In other cases, such as gene body methylation, the regulatory effect is not completely understood, but maybe generated as a footprint of post-transcriptional gene silencing. During the silencing process not only are 21mer siRNA generated but 24mer heterochromatic (hc)-siRNAs can also be generated. These hc-siRNAs lead *via* the RdDM process to methylate the region homologous to the silencing trigger. Further LncRNA are capable of influencing DNA methylation during phases of abiotic stress (Urquiaga et al.).

As the origin of most small RNAs is from repetitive DNA elements and retrotransposons, it is obvious that any environmental change that might lead to transcriptional reactivation of these elements has the potential to change the DNA methylation pattern.

The presented Research Topic contains results produced with the model plants *Arabidopsis thaliana*, presented by Laanen et al. and Paul et al.. They explain the effect of Gamma radiation (Laanen et al.) on the DNA methylation landscape followed over multiple generation. Although abiotic stress usually applies to environmental conditions on our planet, we included also a study investigating epigenetic effects during spaceflight, which should be considered in the context of long term plans for growing plants in space or on other planets.

Therefore, for many environmental changes, differentially methylated genomic areas or sites are described. In some cases, these changes are affecting nearby genes and can cause changes in the phenotype. Although many factors involved in the molecular mechanism of DNA methylation pattern formation are identified, the complex interplay of environmentally induced DNA methylation change and phenotypic change is not always easy to address.

In the present Research Topic, results are presented for monocotyledonous species of economic and ecologic importance, such as barley (*Hordeum vulgare*) (Konate et al.), maize (*Zea mays*) (Madzima et al.) and common reed (*Phragmites australis*) (Wang et al.). In the review by Gallo-Franco et al., rice (*Oryza sativa*) was taken as model to discuss the plant epigenetic response to Aluminum toxicity.

In addition, a good selection of results is provided also for dicotyledon plants. This includes the study of the consequences of cold stress on the methylome of Tartary buckwheat (*Fagopyrum tataricum*), presented by Song et al., and the effect of UV-B radiation on the perennial herb *Glechoma longituba*, by Quan et al., where the authors found that strong UV radiation can influence the plant foraging proprieties. Another study involving a perennial plant includes sweet cherry trees (*Prunus avium*) and investigates the effect of low temperatures on the dormancy of flower buds (Rothkegel et al.).

Finally, in the paper by Srikant and Drost, the epigenetic effects of abiotic stresses are discussed and analyzed in the context of plant adaptation to stresses. The authors hypothesized that plants dynamically integrate physiological, epigenetic and genetic responses to reduce or buffer negative effects on fitness during the adaptation to a changing environment.

Collectively, this collection highlights the relevance of epigenetic response to abiotic stresses in plants in relation to develop new strategies for plant improvement, and to study mechanisms of plant adaptation and evolution. We believe that this selection of works will contribute to clarify the role of epigenetic in plants and can be of inspiration for future works in the same field.

## Author Contributions

MK, HJ, MC, and FJ wrote the editorial.

## Conflict of Interest

The authors declare that the research was conducted in the absence of any commercial or financial relationships that could be construed as a potential conflict of interest.

## Publisher's Note

All claims expressed in this article are solely those of the authors and do not necessarily represent those of their affiliated organizations, or those of the publisher, the editors and the reviewers. Any product that may be evaluated in this article, or claim that may be made by its manufacturer, is not guaranteed or endorsed by the publisher.

